# Mitochondrial Dynamics: A Potential Therapeutic Target for Ischemic Stroke

**DOI:** 10.3389/fnagi.2021.721428

**Published:** 2021-09-07

**Authors:** Xiangyue Zhou, Hanmin Chen, Ling Wang, Cameron Lenahan, Lifei Lian, Yibo Ou, Yue He

**Affiliations:** ^1^Department of Neurosurgery, Tongji Hospital, Tongji Medical College, Huazhong University of Science and Technology, Wuhan, China; ^2^Department of Operating Room, Tongji Hospital, Tongji Medical College, Huazhong University of Science and Technology, Wuhan, China; ^3^Department of Biomedical Sciences, Burrell College of Osteopathic Medicine, Las Cruces, NM, United States; ^4^Department of Neurology, Tongji Hospital, Tongji Medical College, Huazhong University of Science and Technology, Wuhan, China

**Keywords:** energy metabolism, ischemic stroke, molecular mechanisms, mitochondrial dynamics, therapeutic target

## Abstract

Stroke is one of the leading causes of death and disability worldwide. Brain injury after ischemic stroke involves multiple pathophysiological mechanisms, such as oxidative stress, mitochondrial dysfunction, excitotoxicity, calcium overload, neuroinflammation, neuronal apoptosis, and blood-brain barrier (BBB) disruption. All of these factors are associated with dysfunctional energy metabolism after stroke. Mitochondria are organelles that provide adenosine triphosphate (ATP) to the cell through oxidative phosphorylation. Mitochondrial dynamics means that the mitochondria are constantly changing and that they maintain the normal physiological functions of the cell through continuous division and fusion. Mitochondrial dynamics are closely associated with various pathophysiological mechanisms of post-stroke brain injury. In this review, we will discuss the role of the molecular mechanisms of mitochondrial dynamics in energy metabolism after ischemic stroke, as well as new strategies to restore energy homeostasis and neural function. Through this, we hope to uncover new therapeutic targets for the treatment of ischemic stroke.

## Introduction

Stroke is an acute cerebrovascular disease resulting in cerebral blood circulation disorders due to the sudden rupture or occlusion of blood vessels in the brain. Stroke is associated with high morbidity, mortality, and rates of disability (GBD 2016 Stroke Collaborators, 2019), and can be classified as either ischemic or hemorrhagic. Currently, stroke has become the second leading cause of death globally (Lindsay et al., 2019), and is the primary cause of death in China (Wang Y. et al., 2020). It has been a difficult endeavor to save more lives and improve neurological recovery after stroke. As such, this challenge emphasizes the growing need for therapeutic agents that can mitigate brain injury and promote neurological recovery after stroke.

Energy metabolism is an important basis for cellular function, as it is the process by which cells utilize nutrient substances, such as sugars and fats, and produce adenosine triphosphate (ATP). Additionally, ATP is broadly used in cellular activities, and is necessary for ensuring a normal cell lifespan. Mitochondria, which are commonly considered the powerhouse of the cell, are a major site of oxidative metabolism in eukaryotes, and are where sugars, fats, and amino acids are ultimately oxidized to release energy (Cardoso et al., 2010). The state of cellular energy metabolism is closely associated with mitochondrial dynamics, which refers to the dynamic process of mitochondrial fusion and division. Mitochondria maintain a steady state in the mitochondrial network through continuous fusion-division, thus maintaining the normal physiological function of cells (Dorn and Kitsis, 2015). Mitochondrial dynamics are involved in the formation and regulation of mitochondrial permeability transition pores (MPTPs), reactive oxygen species (ROS), and neuronal apoptosis (Roy et al., 2015). Mitochondrial dynamics can affect energy metabolism and post-stroke neuronal function by regulating the number, morphology, and function of mitochondria.

To identify potential interventional targets and novel diagnostic methods, it is crucial to understand the molecular mechanisms, especially those of mitochondrial dynamics after ischemic stroke. Herein, we will discuss the role of mitochondrial dynamics, as well as the energy metabolism involved in ischemic stroke. Moreover, an improved understanding of how mitochondrial dynamics affect energy metabolism will provide opportunities for the development of new therapeutic strategies targeting mitochondrial fusion and division after ischemic stroke.

## Mitochondrial Dynamics and Energy Metabolism in the Brain

Cell energy metabolism refers to the metabolic pathway of ATP synthesis associated with nicotinamide adenine dinucleotide (NADH) turnover (Rigoulet et al., 2020). This pathway mainly includes the decompositional metabolism of sugar (aerobic oxidation, glycolysis, and phosphate sugar pathway), the tricarboxylic acid (TCA) cycle, fatty acid oxidation and synthesis, amino acid metabolism, and vitamin metabolism. Mitochondria are “energy factories” in eukaryotic cells, and are key sites of oxidative phosphorylation. Mitochondria are organelles that are present in most cells and are coated by two layers of membrane. Mitochondria can be divided into four functional regions: the outer mitochondrial membrane (OMM), intermembrane space (IMS), inner mitochondrial membrane (IMM), and mitochondrial matrix (listed in order from outside to inside). The proton concentration gradient originating from the electron transport chain in the IMM drives ATP generation (Scheffler, 2001). Moreover, mitochondria are highly mobile. Mitochondrial dynamics include fusion, division, selective degradation, and transport processes. Dynamic changes in mitochondria are important for immunity, apoptosis, and the cell cycle. These dynamic transformations are mainly mediated by large GTPases that belong to the dynamin family (Tilokani et al., 2018). In addition to generating energy, mitochondria can also drive cell dysfunction or death either passively (through ROS toxicity) or actively (through programed necrosis and apoptosis). Mitochondrial division and fusion play central roles in these processes (Dorn and Kitsis, 2015).

### Mitochondrial Dynamics

**FIGURE 1:**
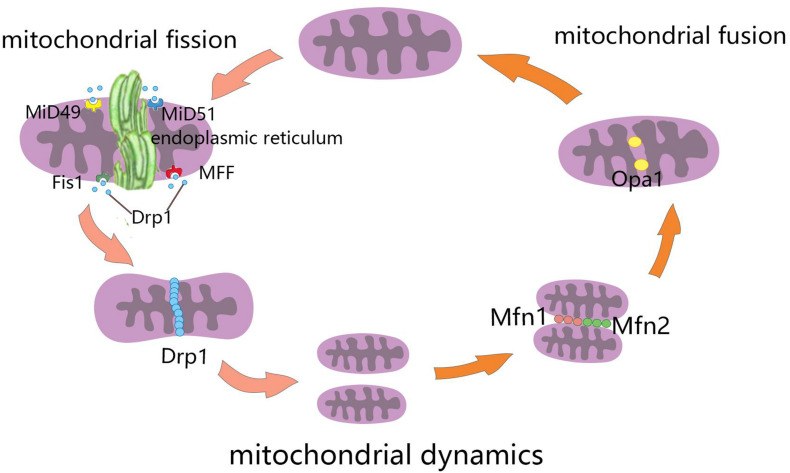
Schematic diagram of mitochondrial dynamics. Mitochondrial fission is the division of mitochondria into two smaller mitochondria, and the dynamin-related GTPase Drp1 plays a crucial role in this process. Drp1 was recruited through four single-way transmembrane Drp1 receptors anchored on the outer mitochondrial membrane (OMM): mitochondrial fission factors (Mff), mitochondrial kinetic proteins 49 and 51(MiD49 and MiD51), and Fis1. Mitochondrial fusion refers to the merging of two mitochondria into one. Mitofusin1 (Mfn1) and mitofusin2 (Mfn2) mediate mitochondrial outer membrane fusion, and optic atrophy protein 1 (Opa1) mediates the fusion of mitochondrial inner membrane.

Mitochondrial fusion refers to the merging of two mitochondria into a single mitochondrion (Figure 1). Because mitochondria have two layers of membrane, the process of mitochondrial fusion consists of outer membrane fusion and inner membrane fusion (Table 1). The tether is a physical connection between the two mitochondrial outer membranes, and is a prerequisite for actual membrane fusion (Koshiba et al., 2004). Mitofusin1 (Mfn1) and Mfn2 mediate fusion of the OMM, and optic atrophy protein 1 (Opa1) mediates fusion of the IMM (Chiurazzi et al., 2020). The overexpression of Mfn2 could increase mitochondrial fusion (Qin et al., 2020). However, the absence of Mfn1, Mfn2 (Hoppins, 2014), or Opa1 can lead to mitochondrial fragmentation (Song et al., 2007). Mitochondrial fusion can also facilitate the exchange of matrix contents among mitochondria through brief contact without resulting in a morphological merge. This is described as kiss-and-run fusion events (Chan, 2020). Mitochondrial fusion, and the material exchanged between mitochondria, optimize mitochondrial function and avoid damage accumulation due to mutations in mitochondrial DNA aging (Westermann, 2010; Chen et al., 2011; Cohen and Tareste, 2018).

**TABLE 1:** Mitochondrial dynamics related proteins.

Mitochondrial dynamics		Proteins	References
Mitochondrial fusion	OMM fusion	Mfn1 Mfn2	Sokoloff et al., 1977; Bruick, 2000; Sowter et al., 2001; Falkowska et al., 2015; Um and Yun, 2017; Cheng et al., 2018; Rodger et al., 2018; Williams and Ding, 2018
	IMM fusion	Opa1	
Mitochondrial fission	Cytoplasm	Drp1	Herrero-Mendez et al., 2009; Belanger et al., 2011; Lunt and Vander Heiden, 2011; Nortley and Attwell, 2017; Magistretti and Allaman, 2018; Bordone et al., 2019
	Mitochondria	Mff MiD49 MiD51 Fis1	

*OMM, outer mitochondrial membrane; IMM, inner mitochondrial membrane; Mfn1, mitofusin1; Mfn2, mitofusin2; Opa1, optic atrophy protein 1; Drp1, dynamin-related protein 1; Mff, mitochondrial fission factors; MiD49, mitochondrial kinetic proteins 49; MiD51, mitochondrial kinetic proteins 51; Fis1, mitochondrial fission protein 1.*

Mitochondrial fission is the division of a mitochondrion into two smaller mitochondria (Figure 1), and dynamin-related GTPase1 (Drp1) plays a crucial role in this process (Table 1). Drp1 is recruited through four single-way transmembrane Drp1 receptors anchored on the OMM: mitochondrial fission factors (Mffs), mitochondrial kinetic proteins 49 and 51(MiD49 and MiD51), and Fis1 (Pagliuso et al., 2018). Mitochondrial fission usually occurs at the endoplasmic reticulum (ER)-mitochondrial contact site (Lewis et al., 2016). Additionally, the contraction of mitochondria is Drp1-independent, and ER tubules are more important in defining the position of mitochondrial fission sites (Friedman et al., 2011). However, mitochondrial fusion and fission are colocalized in ER membrane contact sites (MCSs; Guo et al., 2018). Therefore, mitochondrial fission and fusion could be regulated by controlling certain enzyme-like nodes on the ER MCS (Abrisch et al., 2020). Mitochondrial fission can irreparably fragment mitochondria, remove organelles to maintain mitochondria quality, and protect the normal function of the mitochondrial network (Nunnari, 2007).

Mitochondrial dynamics can regulate mitochondrial morphology, promote mitochondrial substance exchange, maintain mitochondrial DNA and inheritance, and eliminate damaged mitochondria. Normal regulation of mitochondrial dynamics is important in maintaining regular cell activity. Dysregulation of Mitochondrial dynamics plays an important role in driving cell death. Mitochondrial dynamics also play a role in necroptosis. The mitochondrial phosphatase, PGAM5, dephosphorylates Drp1Ser-637, leading to increased ROS and mitochondrial division, as well as promotion of necroptosis (Wang et al., 2012; Yu et al., 2020). When the energy supply is decreased, the increased levels of ADP and AMP can promote mitochondrial division and induce autophagy by activating MiD51 and Mff, respectively (Ducommun et al., 2015; Toyama et al., 2016). In addition, the reduction in Opa1 leads to an increase in autophagy (White et al., 2009). Mitochondrial fission is a necessary step in apoptosis (Xie et al., 2018). Apoptosis is a unique and important mode of programed cell death (Elmore, 2007) that maintains the number of cells in tissues, and it functions as a defense mechanism in the immune response (Norbury and Hickson, 2001). Drp1 can promote Bax translocation to the mitochondria (Montessuit et al., 2010), change the permeability of the mitochondrial membrane, release cytochrome C (Cyt-c), cause a cascade reaction, and lead to apoptosis. Mitochondrial fusion can protect cells from apoptosis (Bueler, 2010). Opa1 maintains the stability of mitochondrial cristae (Varanita et al., 2015) and Mfn2 interferes with Bax translocation by promoting mitochondrial fusion (Neuspiel et al., 2005). However, both Drp1 and Opa1 can prevent apoptosis. A variety of cell death patterns, such as apoptosis, necrosis, phagoptosis, and autophagy, form a complex network with different molecular mechanisms after stroke (Fricker et al., 2018), which suggests that mitochondrial dynamics may play an important role in cell death after stroke. However, in this review we mainly focused on the molecular mechanisms of mitochondrial dynamics in apoptosis after ischemic stroke.

In addition, mitophagy is a defensive mechanism that selectively removes damaged or unnecessary mitochondria *via* autophagy, which plays an important role in maintaining mitochondrial quality control and homeostasis (Gustafsson and Dorn, 2019). Mitophagy is mediated by autophagy-related proteins. Autophagy-related proteins specifically recognize and bind to functionally defective mitochondria so that they become fused with lysosomes to complete the degradation of damaged organelles and proteins (Gustafsson and Dorn, 2019). Mitophagy is closely associated with many functions and physiological processes in cells, such as cell differentiation and development, cell programing, cell death, and the immune response (Um and Yun, 2017). Molecular mechanisms of mitophagy involve PTEN-induced putative kinase 1(PINK1)/Parkin, BCL2/adenovirus E1B 19kDa-interacting protein 3 (BNIP3)/BCL2/adenovirus E1B 19kDa-interacting protein 3-like (NIX), FUN14 domain containing 1 (FUNDC1), BCL2-like 13(BCL2L13), FK506-Binding Protein 8 (FKBP8), and Cardiolipin (Rodger et al., 2018; Williams and Ding, 2018). BNIP3/NIX is associated with hypoxia-induced mitophagy, and the levels of BNIP3 and NIX are upregulated by hypoxia-inducible factor (HIF-1α) transcription (Bruick, 2000; Sowter et al., 2001), suggesting that this pathway may be involved in the brain damage that occurs as a result of the hypoxic ischemia that manifests after stroke.

### Energy Metabolism in the Brain

#### Energy Metabolism in the Brain Under Normal Physiological Conditions

**FIGURE 2:**
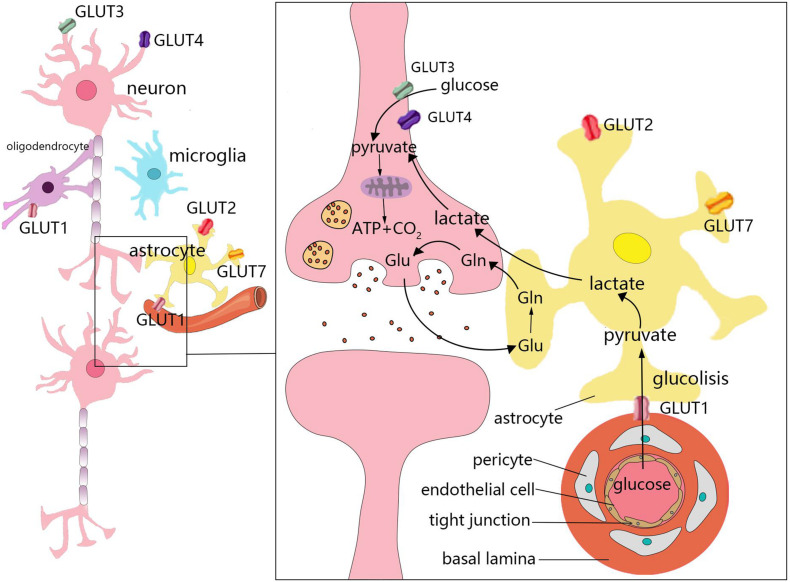
Energy metabolism in the brain. The uptake and utilization of glucose by cells in the brain requires the neurovascular unit, which is composed of brain capillary endothelial cells, pericytes, astrocytes, oligodendrocytes, microglia, and neurons. Glucose enters cells through specific glucose transporters (GLUTs), such as GLUT1, GLUT2, and GLUT7 in astrocytes, GLUT1 in oligodendrocytes, and GLUT3 and GLUT4 in neurons. Numerous metabolic intermediates formed by glucose in the brain can also be oxidized to produce energy, such as lactate, pyruvate, and glutamate. The dynamic regulatory mechanism of lactic acid metabolism between astrocytes and neurons is known as the astrocyte–neuron lactate shuttle. Neurons release glutamate to stimulate glucose uptake by astrocytes. Astrocytes produce lactic acid by aerobic glycolysis in the cytoplasm and then transport to neurons.

The weight of the brain accounts for only 2% of total body weight. However, the brain accounts for 25 and 20% of glucose and oxygen consumption in the body, respectively (Sokoloff et al., 1977). Under aerobic conditions, ATP in brain cells is primarily derived from glucose that is consumed in the TCA cycle occurring in the mitochondria for oxidative phosphorylation. Moreover, the uptake and utilization of glucose by cells in the brain are associated with specific features. This process requires several cell types, which comprise the neurovascular unit, to coordinate and complete it. The neurovascular unit is composed of brain capillary endothelial cells, pericytes, astrocytes, oligodendrocytes, microglia, and neurons (Cheng et al., 2018; Figure 2). Glucose enters cells through specific glucose transporters (GLUTs), such as GLUT1, GLUT2, and GLUT7 in astrocytes, GLUT1 in oligodendrocytes, and GLUT3 and GLUT4 in neurons. Additionally, glucose is phosphorylated by hexokinase to produce glucose-6-phosphate (Falkowska et al., 2015). Glucose 6-phosphate can be processed through various metabolic pathways (e.g., glycolysis, pentose phosphate pathway, and glycogenesis). Numerous metabolic intermediates formed by glucose in the brain, such as lactate, pyruvate, glutamate, or acetate, can also be utilized to produce energy (Belanger et al., 2011).

In the brain, different cell types have different metabolic characteristics. There is dynamic regulation between astrocytes and neurons known as the astrocyte–neuron lactate shuttle (Bordone et al., 2019; Figure 2). Neurons release glutamate to stimulate astrocytes to take up glucose (Magistretti and Allaman, 2018). Due to the low activity of pyruvate dehydrogenase in astrocytes, it is easier to produce lactic acid *via* aerobic glycolysis in the cytoplasm, which is also known as the Warburg effect in cancer cells (Lunt and Vander Heiden, 2011; Magistretti and Allaman, 2018). Astrocytes play a significant role in regulating the energy supply of the brain, but it remains unclear whether the lactic acid produced by astrocytes is fuel for neurons (Nortley and Attwell, 2017). Neurons lack fructose 6-phosphate-2-kinase/fructose-2,6- bisphosphatase-3(Pfkfb3), thus demonstrating a slower rate of glycolysis than other cells. However, neurons can efficiently utilize lactic acid (Herrero-Mendez et al., 2009; Bolanos, 2016). After glucose is transported through the blood-brain barrier (BBB) and the cell membrane, it is transformed into pyruvate *via* anaerobic glycolysis in the cytoplasm, which leads to the generation of 2 mol of ATP. Each mole of glucose pyruvate is subsequently transported to the mitochondria, and is then converted into acetyl coenzyme A, which then participates in the TCA cycle. During the subsequent steps of the TCA cycle, oxidative phosphorylation produces an additional 30 mol of ATP per mole of glucose. This process is closely associated with the TCA cycle, electron transfer in the respiratory chain, oxygen consumption, and the production of carbon dioxide and water. In addition to basic cell activities, the energy produced by various cells in the brain is mainly used to maintain and restore ion gradients that are dissipated by signaling processes (e.g., postsynaptic and action potentials), as well as participate in the uptake and recycling of neurotransmitters (Attwell and Laughlin, 2001).

#### Energy Metabolism in the Brain After Ischemic Stroke

Obviously, ischemic hypoxic brain injury after stroke is closely associated with energy metabolism disorder. However, there are various pathophysiological mechanisms involved in brain damage after stroke, such as oxidative stress, mitochondrial dysfunction, excitotoxicity, calcium overload, neuroinflammation, acidosis, neuronal apoptosis, and BBB disruption (Moskowitz et al., 2010; Sekerdag et al., 2018). The normal supply of glucose and oxygen is cut off during cerebral ischemia, although astrocytes can synthesize and store glycogen, as well as metabolize glycogen into lactic acid to provide energy for neurons (Mergenthaler et al., 2013; Falkowska et al., 2015). When the glucose supply is insufficient, ketone bodies and lactic acid can also become substrates for brain energy metabolism (Sokoloff, 1981). In early ischemia, when synaptic activity disappears, adenosine is released to block presynaptic Ca^2+^ influx and inhibit glutamate release (Hofmeijer and van Putten, 2012). This early inhibition of glutamate release prevents glutamate excitotoxicity. However, if the ischemic period is prolonged, neuronal cells cannot maintain the normal transmembrane concentration gradient, which causes neuronal signal impairment. Conversely, synaptic terminal depolarization releases the neurotransmitter glutamate. Furthermore, the reabsorption of glutamate clearance from the synaptic space leads to glutamic acid accumulation, excessive stimulation of N-methyl-D-aspartate (NMDA) receptors, high levels of calcium influx, mitochondrial depolarization, the release of Cyt-c, and neuronal apoptosis (Harris et al., 2012; Campbell et al., 2019).

After stroke, different regions comprising the stroke lesion will exhibit various metabolic characteristics. The location of the stroke lesion can be divided into three regions according to cerebral blood flow (CBF; Pushie et al., 2018). The ischemic core area is the central region of brain tissue infarct. The ischemic periphery region surrounding the core is divided into two components. One is close to the infarct area. Here, there are a large number of cells undergoing oxidative stress due to the reduced blood supply, but timely reperfusion therapy can save this section of tissue, known as the ischemic penumbra. The ischemic peripheral region is relatively far from the infarct area, and the blood circulation is relatively similar to that of normal tissue. The levels of glucose utilization, ATP, lactic acid, creatine phosphate, and pH varies according to CBF changes (Leigh et al., 2018). The ischemic penumbra cannot be rescued and it expands into the infarct area when CBF decreases beyond a certain threshold (Astrup et al., 1981). Currently, it is an important objective in stroke treatment to identify ischemic penumbral tissue based on PET/CT and related brain metabolism (Sarrafzadeh et al., 2010; Zenonos and Kim, 2010), and to treat it in a timely manner. Due to the different metabolic demands of grey matter (GM) and white matter (WM), the CBF and cerebral blood volume (CBV) thresholds of the damaged region are different (An et al., 2015; Leigh et al., 2018). Moreover, GM is more susceptible to ischemia than WM because GM has a higher CBF, CBV, and apparent diffusion coefficient (ADC), as well as a shorter mean transit time (MTT; Bristow et al., 2005). GM mainly includes neuronal cell bodies, dendrites, and axons, which are used for local information, while WM mainly includes axons, oligodendrocytes, and astrocytes. Neuronal apoptosis or necrosis results from excessive free radical production, calcium overload, and excitotoxicity following ischemia and hypoxia (Radak et al., 2017). The decreased blood supply after stroke leads to the destruction of axonal electrophysiological characteristics and nutritional dysfunction in WM. The effect of glutamate metabolism on astrocytes induces excitotoxicity in oligodendrocytes (Wang et al., 2016). After reperfusion, energy metabolism does not immediately return to baseline as expected. The activation of platelets and complement systems, the release of inflammatory mediators, and neuronal mitochondria in the ischemic penumbra overcompensate for ischemic injury by inducing metabolic pathways, which leads to the excessive release of ROS. All of these factors contribute to neuronal death and brain injury after ischemia-reperfusion (Al-Mufti et al., 2018).

## The Role of Mitochondrial Dynamics in Brain Injury After Ischemic Stroke

### The Molecular Mechanisms of Mitochondrial Dynamics in the Ischemic Stroke

**FIGURE 3:**
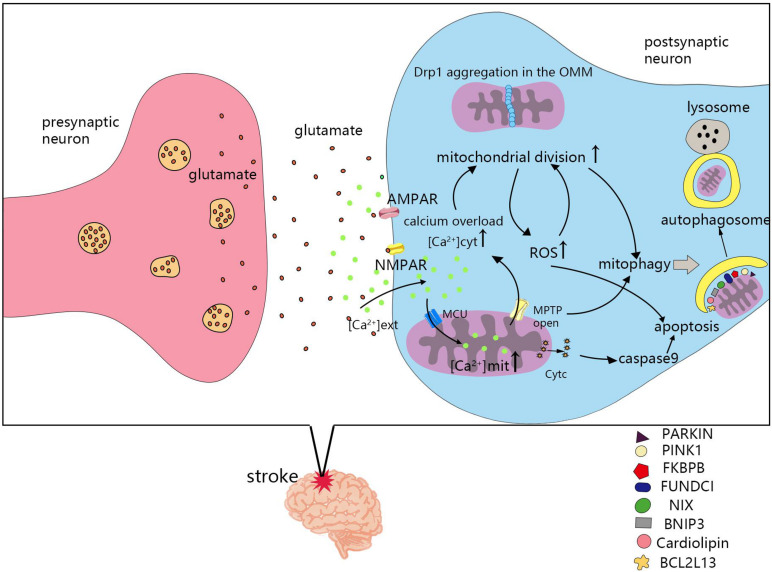
Molecular mechanisms of mitochondrial dynamics in post-ischemic stroke injury. Cerebral ischemia and hypoxia lead to the imbalance of mitochondrial division and fusion, which is related to calcium overload, reactive oxygen species (ROS), mitochondrial permeability transition pores(MPTP), apoptosis, and mitophagy. MCU, mitochondrial calcium uniporter; NMPAR, N-methyl-D-aspartate receptors; AMPAR, α-amino-3-hydroxy-5-methyl-4-isoxazole-propionic acid receptors; [Ca^2+^] ext, calcium ion concentration in extracellular; [Ca^2+^] cyt, calcium ion concentration in cytoplasmic; [Ca^2+^] mit, calcium ion concentration in mitochondria.

The mechanism of mitochondrial division and fusion is complex, and not only affects energy metabolism in cells, but also induces apoptosis. After cerebral ischemia and hypoxia, changes in mitochondrial dynamics also greatly impact the survival of nerve cells. Seventy percent of neuronal energy is used to maintain the sodium and potassium pump on the cell membrane. The ATP supply is insufficient after stroke, which leads to depolarization of the neuronal plasma membrane, release of the excitatory neurotransmitter, glutamate, and causes glutamate excitotoxicity (Doyle et al., 2008). Ischemia induced an increase in glutamate release from neurons and astrocytes, which leads to overstimulation of NMDA and α-amino-3-hydroxy-5-methyl-4-isoxazole-propionic acid (AMPA) receptors. Glutamate excitotoxicity and oxidative stress influence mitochondrial division and fusion, as well as the imbalance in mitochondrial division and fusion, leading to NMDA receptor upregulation and oxidative stress (Nguyen et al., 2011). Next, we will review the molecular mechanisms of mitochondrial dynamics in post-stroke injury (Figure 3).

#### Calcium Overload

Excessive calcium enters cells, activates many calcium-dependent proteases, lipases, and deoxyribonucleases, and leads to cell death (Besancon et al., 2008). The mitochondrial calcium uniporter (MCU), a calcium transporter in mitochondria, plays a vital role in maintaining intracellular homeostasis by transporting Ca^2+^ from the cytoplasm into the mitochondrial matrix (Zhao et al., 2013; Kwong et al., 2015). Mitochondrial Ca^2+^ controls energy production and metabolism by regulating key enzymes and fatty acid oxidation in the TCA cycle. Calcium overload in the cytoplasm activates calcineurin, and dephosphorylates Drp1 at serine 637, leading to Drp1 accumulation from the cytoplasm to the OMM. Promoting mitochondrial division and ROS production, but inhibiting mitochondrial division by using mitochondrial division inhibitor 1(Mdivi-1), Drp1siRNA, or calcineurin inhibitors shows that mitochondrial morphology is retained, intracellular calcium ions are reduced, and cell death is prevented in cardiac ischemia-reperfusion injury (Sharp et al., 2014). Inhibiting Drp1 can participate in neuroprotection by combating glutamate toxicity *in vitro* and ischemic brain injury *in vitro* (Grohm et al., 2012). Calcium overload also causes mitochondria to release apoptotic factors and induce apoptosis (Pivovarova et al., 2004).

#### Reactive Oxygen Species

Reactive oxygen species and oxidative stress are important causes of tissue damage during cerebral ischemia (Crack and Taylor, 2005). Mitochondria are thought to be the main origin of intracellular ROS (Bayir and Kagan, 2008). Oxygen-free radicals are highly active and have the ability to destroy cellular components (Droge, 2002). Changes in ROS lead to changes in the expression or activity of proteins associated with mitochondrial dynamics that affect mitochondrial fusion and division (Cid-Castro et al., 2018). In general, elevated ROS levels trigger mitochondrial fission. It is worth mentioning that phosphorylation at different sites of Drp1 differentially affects mitochondrial dynamics. The increase in ROS promotes Drp1 activation through Ser616 phosphorylation, which promotes mitochondrial fission (Cho et al., 2012). However, Drp1 serine 637 phosphorylation inhibits mitochondrial fission. The phosphorylation of Drp1 at tyrosines 266, 368, and 449 leads to mitochondrial division and neuronal death (Zhou et al., 2017). The Drp1-mitochondrial fission ROS cycle may play a role in central nervous system (CNS) diseases (da Rosa et al., 2020). There is an interaction between mitochondrial dynamics and ROS production. On one hand, increased mitochondrial division will increase the production of ROS, but inhibition of mitochondrial division can restore ROS levels to normal (Zhang Y. K. et al., 2020). On the other hand, the increase of ROS will promote the activation of Drp1, leading to increased mitochondrial division (Youle and van der Bliek, 2012; Cid-Castro et al., 2018), thereby further increasing the production of ROS, whereas knocking out Drp1 reduces oxidative stress-induced mitochondrial fragmentation (Youle and van der Bliek, 2012). This cyclic reaction will aggravate mitochondrial dysfunction after stroke, increase the level of ROS, and increase brain damage after stroke. After inhibiting ROS levels in a mouse model of stroke, mitochondrial dysfunction, obstruction volume, and neurological deficits were reduced (Hwang et al., 2020). From this perspective, interrupting this cycle by reducing ROS levels or inhibiting mitochondrial division is a key part of stroke treatment. Studies have shown that inhibiting excessive mitochondrial division could protect against neurotoxicity (Rappold et al., 2014). Hyperbaric oxygen (HBO) can also reduce brain damage by regulating Drp1 phosphorylation (Ni et al., 2020). ROS can also activate OMA1 to promote the cleavage of L-Opa1 to S-Opa1, resulting in mitochondrial crest remodeling and Cyt-c release, further resulting in increased apoptosis (Zhang K. et al., 2014).

#### Mitochondrial Permeability Transition Pores

The MPTP is a channel that triggers a sudden increase in the permeability of the mitochondrial inner membrane when there is mitochondrial calcium overload, especially when combined with oxidative or nitrite stress and/or ATP depletion (Bernardi, 1999). When the MPTP is open, it can lead to a rapid increase in the osmotic pressure of the matrix solute, rupture of the OMM, collapse of the mitochondrial membrane potential (ΔΨm), and a reduction in intracellular ATP, which eventually leads to necrotic cell death (Kinnally et al., 2011). The MPTP can also be used as a physiological efflux pathway, and can open transiently (Altschuld et al., 1992). In cyclophilin D-knockout mice, transient activation of the MPTP could protect neurons from cytoplasmic Ca^2+^ overload (Barsukova et al., 2011). Mitochondrial matrix calcium overload is the main mechanism of MPTP opening, and plays an important role in regulating the MPTP (Hurst et al., 2017). Moreover, ROS can also regulate the activity of the MPTP (Halestrap et al., 1997). Hence, the opening of the MPTP is involved in the pathophysiological mechanism after stroke. The infarct size after transient occlusion of the artery can be reduced by the cyclosporine A analog, N-methyl-Val-4-cyclosporin A (Me ValCsA), which blocks the MPTP (Matsumoto et al., 1999). Melatonin and atorvastatin can also block the MPTP and the release of Cyt-c from mitochondria, thereby reducing neuronal apoptosis after ischemia-reperfusion (Andrabi et al., 2004; Song et al., 2014). Treatment of HL-1 cells with Drp-1 inhibitors reduces mitochondrial permeability transition pore sensitivity after cardiac ischaemia-reperfusion (Ong et al., 2010). In Mfn-2-deficient mice, MPTP opening is delayed. Therefore, Mfn-2 participates in the regulation of the MPTP and triggers death in cardiomyocytes (Papanicolaou et al., 2011).

#### Apoptosis

After apoptotic stimulation, apoptotic members of the Bcl-2 protein family, such as Bax and Bak, insert into the mitochondrial outer membrane (Ader et al., 2019) and change the permeability of the OMM, thus mediating the release of apoptosis-related factors, such as Cyt-c, second mitochondria-derived activator of caspases (Smac), and apoptosis-inducing factor (AIF) from mitochondria into the cytoplasmic matrix (Jurgensmeier et al., 1998; Susin et al., 1999; Jeong and Seol, 2008). Apoptosis-related factors in the cytoplasm activate caspase-9, causing cascade reactions, consequently leading to apoptosis (Green and Reed, 1998). However, mitochondrial dynamics is involved in the regulation of apoptosis (Frank et al., 2001). The dominant-negative mutant of Drp1, as well as siRNA-mediated silencing of Drp1, could reduce apoptosis in Q111/0 cells in Huntington’s disease (Costa et al., 2010). By inhibiting Drp1 activation, mitochondrial debris and ROS can be reduced, and neuronal damage can be alleviated (Zhou et al., 2020). Using propofol to treat oxidation-injured neurons could reduce the expression of Fis1 and increase the expression of Mfn1, maintaining normal levels of these two proteins, thus reducing the expression of apoptotic proteins in neurons and alleviating neuronal damage induced by hypoxia (Zhang H. S. et al., 2020). Research has shown that inducing mitochondrial fission *via* activation of Drp1 and Mff results in apoptosis in hippocampal neuronal cells (Zhang C. et al., 2020).

#### Mitophagy

The regulation of mitochondrial autophagy is associated with mitochondrial dynamics (Yoo and Jung, 2018), and is expected to be a new target for ischemic stroke treatment (Guan et al., 2018). In an acute model of mitochondrial damage induced by carbonyl cyanide-m-chlorophenylhydrazone (CCCP), the ubiquitination of mitofusin 1 (Mfn-1) and 2 (Mfn-2) is closely associated with the induction of mitophagy, and the ubiquitination of both proteins is dependent on parkin and PINK1, which play crucial roles in mitophagy (Gegg et al., 2010). In Parkin-null HeLa cells and in HEK293 cells with siRNA-mediated Parkin knockdown, glycoprotein 78 (Gp78) induction of mitophagy was Mfn1-dependent (Fu et al., 2013). In adult cardiac progenitor cells (CPCs), knockdown of either Fundc1 or Bnip3l had no effect on Mfn1/2 protein levels, but led to the activation of DNM1L and the promotion of mitochondrial fission during differentiation (Lampert et al., 2019). In proximal tubule-specific Fundc1-knockout mice, Drp1 is overactivated and leads to mitochondrial division (Wang J. et al., 2020). In traumatic brain injury (TBI) models, the Drp1 inhibitor, Mdivi-1, could inhibit the activation of PTEN-induced putative kinase 1 (PINK1)-Parkin mediated mitophagy (Wu et al., 2018).

### Mitochondrial Dynamics in Different Neuronal Cells After Ischemic Stroke

Mitochondrial dynamics play various roles in different cells after stroke. Because of the length of axons and special energy requirements in neurons, the distribution, movement, and timely fusion and repair of mitochondria are particularly important in maintaining normal neuronal function (Mandal and Drerup, 2019). Oxygen-glucose deprivation (OGD) can induce mitochondrial division, leading to autophagy or apoptosis (Wu et al., 2017). Astrocytic endfeet are enriched in mitochondria-ER contact sites. These sites, as well as mitochondrial fusion, can promote vascular remodeling after brain injury (Gobel et al., 2020). After several hours of OGD, the length of the astrocytic endfeet were shortened, mitochondrial fission was delayed, and the mitochondria were lost (O’Donnell et al., 2016). Increased mitochondrial division in astrocytes during hypoxia may be a possible method of increasing mitochondrial energy production (Quintana et al., 2019). In microglia, the inflammatory response induced by OGD can be improved by inhibiting mitochondrial fission (Zhou et al., 2019b). Mitochondrial division can also promote the production of mitochondrial ROS in microglia by activating NF-κB and mitogen-activated protein kinase (MAPK), and by inducing the expression of proinflammatory mediators (Park et al., 2013). The neurovascular unit is composed of neurons, glial cells, such as astrocytes and microglia, vascular cells, such as endothelial cells and perivascular cells, and basement membrane (Iadecola, 2017). After inducing excessive mitochondrial division, endothelial cell death also increased (Rao et al., 2020). After stroke, changes in mitochondrial dynamics affect the cells of the neurovascular unit, thus destroying the BBB. Using Mdivi-1 to inhibit mitochondrial division can reduce damage to the BBB after TBI (Wu et al., 2018). Oligodendrocytes primarily wrap axons and form insulated myelin structures, which are essential in maximizing the conduction and velocity of the action potential. Unlike neurons, mitochondria are sparsely distributed in oligodendrocytes with poor fluidity, and glutamate activation can promote mitochondrial motility in oligodendrocytes (Rinholm et al., 2016). However, unlike in astrocytes, the use of mitochondrial division inhibitor 1 makes oligodendrocytes sensitive to excitotoxicity and ER stress due to their non-targeted effects, resulting in oxidative stress and apoptosis (Ruiz et al., 2020).

### The Role of Mitochondrial Dynamics in Ischemic Stroke

As mentioned previously, the interaction between calcium overload, ROS production, and the MPTP leads to an increase in mitochondrial fission in ischemic stroke. Although increased mitochondrial fission during hypoxia may increase mitochondrial energy production, which is beneficial in the maintenance of neural function after stroke (Quintana et al., 2019), inducing mitochondrial fission is detrimental to neurons (Zhang C. et al., 2020). However, inhibiting Drp1 to restore the balance of mitochondrial fission and fusion could reduce Bax oligomerization and the release of apoptotic factors after ischemic stroke, thereby reducing the volume of cerebral infarction (Zhao et al., 2014). Exercise preconditioning could promote mitochondrial fusion after cerebral ischemia by up-regulating OPA1, thus reducing cerebral edema and improving neurologic function in ischemic stroke (Zhang L. et al., 2014). Mitochondrial fission can clear damaged mitochondria through autophagy, but excessive mitochondrial division affects the normal function of mitochondria so that the production of ATP is reduced (Wai and Langer, 2016; Sprenger and Langer, 2019). Dysfunction of mitochondrial dynamics can lead to obstacles in mitochondrial distribution and transport in neurons, which can lead to insufficient local energy in neurons (Sheng, 2014). Excessive mitochondrial fission also affects intracellular calcium homeostasis and exacerbates excitotoxicity (Wang et al., 2015). However, mitochondrial fusion can repair damaged mitochondria (Cohen and Tareste, 2018) and produce additional energy by upregulating the activity of ATP synthase through the remodeling of mitochondrial cristae (Gomes et al., 2011). Therefore, inhibiting excessive mitochondrial fission, properly promoting mitochondrial fusion, and restoring the balance of mitochondrial dynamics are beneficial in recovery after ischemic stroke.

## Treatments Targeting Mitochondrial Dynamics After Ischemic Stroke

The clinical treatment of ischemic stroke primarily includes acute stage treatment and subacute stage treatment. The acute stage occurs within 4.5 h. If patients meet admission criteria and have no thrombolytic contraindications, thrombolytic therapy is recommended (Powers et al., 2018). Currently, great progress has been made in endovascular treatment, such as mechanical thrombectomy, which has played a great role in the treatment of stroke (Prabhakaran et al., 2015). However, these treatments still have limitations, such as a strict time window for thrombolytic therapy, and there is a certain risk of complications, such as hemorrhagic transformation (Yaghi et al., 2017). Therefore, it is necessary to find new therapeutic targets and to develop corresponding drugs. The mitochondrial dynamics are closely associated with energy metabolism after stroke and pathophysiological mechanisms, such as ROS, apoptosis, and autophagy. Therefore, the molecular mechanisms associated with mitochondrial dynamics may represent new directions in ischemic stroke treatment. However, the excessive mitochondrial division plays an essential role in ischemic stroke. Therefore, an emphasis should be placed on inhibiting excessive mitochondrial fission and restoring the balance of mitochondrial dynamics when using mitochondrial dynamics as a focal point in the treatment of ischemic stroke (Table 2).

**TABLE 2:** Drugs targeting mitochondrial dynamics after ischemic stroke.

Target	Treatment	Mechanism	Effect	References
Inhibit mitochondrial fission	Atractylenolide III	Reduce Drp1 phosphorylation and translocation by inhibiting the JAK2/STAT3 pathway	Attenuate cerebral edema and neurological deficits	Zhou et al., 2019a
	AG490			
	miR-7 mimics	Repress α-synuclein, a protein that induces mitochondrial fragmentation	Reduce the post-ischemic lesion volume, accelerate motor function recovery, and ameliorate motor and cognitive deficits in mice	Kim et al., 2018
	Nitric Oxide Synthase 3 (NOS3) inhibition	Regulate Miro-2 levels and prevent mitochondrial division,	Promote axonal functional recovery	Bastian et al., 2018
	peptide P110	Inhibit the interaction between Drp1 and Fis1	Increase neuronal cell viability by reducing apoptosis and autophagic cell death	Qi et al., 2013
	photobiomodulation therapy	Inhibit hypoxic-ischemic-induced mitochondrial fragmentation	Reduce neuronal apoptosis in neonatal hypoxic-ischemic encephalopathy	Tucker et al., 2018
Promote mitochondrial fusion	Melatonin	Upregulate Opa1 expression by activating the Yap-Hippo pathway	Reduce infarct size and cerebral reperfusion stress, inhibit neuronal death	Wei et al., 2019
Restore the balance of mitochondrial dynamics	B355252	Restore Mfn2, p-Drp1, and Fis1 levels	Decrease the mitochondrial membrane potential and ROS, reduce the autophagy induction	Chimeh et al., 2018
	deletion of Nurr1	Inhibit Fis1/Drp1 expression, reverse the levels of Mfn2 and Opa1	Reduce neuronal death	Zhang and Yu, 2018
	subcutaneous injection of G-CSF	Reduce levels of Beclin-1, Bax, Bak, and Drp1, upregulate Opa1	Reduce apoptosis, and protect neurons in cerebral ischemia	Modi et al., 2020
Others	Mitochondrial transplantation	Transfer of exogenous mitochondria through local or systemic intra-arterial injections	Reduce brain damage, cell death, and motor function in MCAO rats	Huang et al., 2016; Chang et al., 2019

*B355252: 4-chloro-*N*-(naphthalen-1-ylmethyl)-5-(3-(piperazin-1-yl) phenoxy) thiophene-2-sulfon-amiden.*

*NOS, Nitric oxide synthase; G-CSF, granulocyte-colony stimulating factor.*

Atractylenolide III and AG490 (an inhibitor of jak2) therapy in middle cerebral artery occlusion (MCAO) mice could reduce Drp1 phosphorylation and translocation, as well as and mitochondrial division though the JAK2/STAT3 pathway, thereby attenuating cerebral edema and neurological deficits (Zhou et al., 2019a). Melatonin upregulates Opa1 expression by activating the Yap–Hippo pathway, thereby promoting mitochondrial fusion, reducing infarct size, inhibiting neuronal death, and reducing cerebral reperfusion stress (Wei et al., 2019). Opa1, Mfn2, p-Drp1, and FIS1 were decreased to varying degrees in the mouse model of CoCl_2_-induced cerebral hypoxia. However, 4-chloro-*N*-(naphthalen-1-ylmethyl)-5-(3-(piperazin-1-yl) phenoxy) thiophene-2-sulfon-amide (B355252) treatment could restore Mfn2, p-Drp1, and Fis1 levels, maintain mitochondrial stability, restore mitochondrial membrane potential, and reduce ROS (Chimeh et al., 2018). Injection of miR-7 mimic oligonucleotide after cerebral ischemia could repress α-synuclein, a protein that induces mitochondrial fragmentation, oxidative stress, autophagy, and the promotion of neuronal cell death, thus reducing brain injury after stroke (Kim et al., 2018). Nitric oxide synthase 3 (NOS3) inhibition regulates Miro-2 levels, prevents mitochondrial division, and promotes axonal functional recovery by protecting mitochondrial structure and movement (Bastian et al., 2018). Fis1 and Drp1 are increased, and both Mfn2 and Opa1 are downregulated after cerebral ischemia-reperfusion, indicating that cerebral ischemia-reperfusion induces excessive mitochondrial division and prevents mitochondrial fusion. However, deletion of Nurr1 could inhibit Fis1/Drp1 expression, reverse the levels of Mfn2 and Opa1, correct the imbalance in mitochondrial division and fusion, and reduce neuronal death (Zhang and Yu, 2018). In a mouse model of bilateral common carotid artery occlusion, subcutaneous injection of granulocyte-colony stimulating factor (G-CSF) was used as treatment. G-CSF reportedly reduced the levels of the autophagy marker, Beclin-1, and the proapoptotic proteins, Bax, Bak, and Drp1, but upregulated the mitochondrial fusion protein, Opa1. Therefore, G-CSF can maintain cellular homeostasis by maintaining the stability of mitochondrial dynamics, reducing apoptosis, and protecting neurons in cerebral ischemia (Modi et al., 2020).

However, mitochondrial fission is necessary for maintaining normal cellular function, and it is unrealistic to inhibit mitochondrial fission completely. Some studies have focused on the role of the interaction between Drp1 and its recruitment molecules, such as Mff (Kornfeld et al., 2018) and Fis1 (Qi et al., 2013), in physiological and pathological mitochondrial fission. One study showed that peptide P259-mediated inhibition of the Drp1-Mff interaction could influence normal mitochondrial morphology and basic function under physiological conditions (Kornfeld et al., 2018). The peptide P110 could inhibit the interaction between Drp1 and Fis1, thus reducing excessive mitochondrial fission under pathological conditions without affecting physiological fission (Qi et al., 2013). Moreover, some biomarkers, such as flavin adenine dinucleotide fluorescence (Berndt et al., 2020), can be used as early markers of mitochondrial damage during brain hypoxia, which helps in studying the optimal time to administer drugs targeting mitochondria. There are also some new therapies, such as photobiomodulation therapy, which can inhibit hypoxic-ischemia-induced mitochondrial fragmentation, alleviate mitochondrial dysfunction and oxidative stress, and ultimately reduce neuronal apoptosis in neonatal hypoxic-ischemic encephalopathy (Tucker et al., 2018). Mitochondrial transplantation is also on the rise, and the transfer of exogenous mitochondria through local or systemic intra-arterial injections reduces brain damage, cell death, and motor function in MCAO rats (Huang et al., 2016; Chang et al., 2019).

## Conclusion and Perspective

Energy metabolism after ischemic stroke is closely associated with mitochondrial dynamics. Targeting mitochondrial dynamics-related molecular mechanisms and associated processes, such as calcium overload, ROS, MPTP, apoptosis, and mitophagy, can alleviate brain injury after stroke by improving mitochondrial function and its effect on energy metabolism. This review shows that mitochondrial dynamics play essential roles in pathophysiological changes after stroke. However, the related studies of mitochondrial dynamics mainly focus on cardiac ischemia-reperfusion and neurodegenerative diseases. Mitochondrial dynamics should also be studied intensively in ischemic stroke to understand the specific regulatory mechanisms of mitochondrial dynamics on the clinical manifestations and prognosis of stroke. The clinical application and long-term prognosis of stroke patients are also worthy of further study. In addition, most drug-related effects have only been examined in laboratory studies. There have been few studies on clinical applications. Whether mitochondrial dynamics-targeted drugs could improve a stroke patient’s condition and prognosis in the clinic is a challenge to be explored in subsequent drug studies.

## Author Contributions

XZ and YH conceived the main outline. XZ wrote the manuscript. LW and HC made the figures. CL, LL, and YO took charge for the manuscript revision in English. YH participated in the correction and finally reviewed of this manuscript. All authors contributed to the article and approved the submitted version.

## Conflict of Interest

The authors declare that the research was conducted in the absence of any commercial or financial relationships that could be construed as a potential conflict of interest.

## Publisher’s Note

All claims expressed in this article are solely those of the authors and do not necessarily represent those of their affiliated organizations, or those of the publisher, the editors and the reviewers. Any product that may be evaluated in this article, or claim that may be made by its manufacturer, is not guaranteed or endorsed by the publisher.

## References

[B1] Abrisch R. G. Gumbin S. C. Wisniewski B. T. Lackner L. L. Voeltz G. K. (2020). Fission and fusion machineries converge at ER contact sites to regulate mitochondrial morphology. *J. Cell Biol.* 219:e201911122. 10.1083/jcb.201911122 32328629 PMC7147108

[B2] Ader N. R. Hoffmann P. C. Ganeva I. Borgeaud A. C. Wang C. Youle R. J. (2019). Molecular and topological reorganizations in mitochondrial architecture interplay during Bax-mediated steps of apoptosis. *eLife* 8:e40712. 10.7554/eLife.40712 30714902 PMC6361589

[B3] Al-Mufti F. Amuluru K. Roth W. Nuoman R. El-Ghanem M. Meyers P. M. (2018). Cerebral ischemic reperfusion injury following recanalization of large vessel occlusions. *Neurosurgery* 82 781–789. 10.1093/neuros/nyx341 28973661

[B4] Altschuld R. A. Hohl C. M. Castillo L. C. Garleb A. A. Starling R. C. Brierley G. P. (1992). Cyclosporin inhibits mitochondrial calcium efflux in isolated adult rat ventricular cardiomyocytes. *Am. J. Physiol.* 262 H1699–H1704. 10.1152/ajpheart.1992.262.6.H1699 1377876

[B5] An H. Ford A. L. Chen Y. Zhu H. Ponisio R. Kumar G. (2015). Defining the ischemic penumbra using magnetic resonance oxygen metabolic index. *Stroke* 46 982–988. 10.1161/Str.0000000000000063 25721017 PMC4533116

[B6] Andrabi S. A. Sayeed I. Siemen D. Wolf G. Horn T. F. (2004). Direct inhibition of the mitochondrial permeability transition pore: a possible mechanism responsible for anti-apoptotic effects of melatonin. *FASEB J.* 18 869–871. 10.1096/fj.03-1031fje 15033929

[B7] Astrup J. Siesjo B. K. Symon L. (1981). Thresholds in cerebral ischemia – the ischemic penumbra. *Stroke* 12 723–725. 10.1161/01.str.12.6.723 6272455

[B8] Attwell D. Laughlin S. B. (2001). An energy budget for signaling in the grey matter of the brain. *J. Cereb. Blood Flow Metab.* 21 1133–1145. 10.1097/00004647-200110000-00001 11598490

[B9] Barsukova A. Komarov A. Hajnoczky G. Bernardi P. Bourdette D. Forte M. (2011). Activation of the mitochondrial permeability transition pore modulates Ca2+ responses to physiological stimuli in adult neurons. *Eur. J. Neurosci.* 33 831–842. 10.1111/j.1460-9568.2010.07576.x 21255127 PMC3183752

[B10] Bastian C. Zaleski J. Stahon K. Parr B. McCray A. Day J. (2018). NOS3 inhibition confers post-ischemic protection to young and aging white matter integrity by conserving mitochondrial dynamics and Miro-2 levels. *J. Neurosci.* 38 6247–6266. 10.1523/JNEUROSCI.3017-17.2018 29891729 PMC6041791

[B11] Bayir H. Kagan V. E. (2008). Bench-to-bedside review: mitochondrial injury, oxidative stress and apoptosis–there is nothing more practical than a good theory. *Crit. Care* 12:206. 10.1186/cc6779 18341705 PMC2374589

[B12] Belanger M. Allaman I. Magistretti P. J. (2011). Brain energy metabolism: focus on astrocyte-neuron metabolic cooperation. *Cell Metab.* 14 724–738. 10.1016/j.cmet.2011.08.016 22152301

[B13] Bernardi P. (1999). Mitochondrial transport of cations: channels, exchangers, and permeability transition. *Physiol. Rev.* 79 1127–1155. 10.1152/physrev.1999.79.4.1127 10508231

[B14] Berndt N. Kovacs R. Rosner J. Wallach I. Dreier J. P. Liotta A. (2020). Flavin adenine dinucleotide fluorescence as an early marker of mitochondrial impairment during brain hypoxia. *Int. J. Mol. Sci.* 21:3977. 10.3390/ijms21113977 32492921 PMC7312830

[B15] Besancon E. Guo S. Lok J. Tymianski M. Lo E. H. (2008). Beyond NMDA and AMPA glutamate receptors: emerging mechanisms for ionic imbalance and cell death in stroke. *Trends Pharmacol. Sci.* 29 268–275. 10.1016/j.tips.2008.02.003 18384889

[B16] Bolanos J. P. (2016). Bioenergetics and redox adaptations of astrocytes to neuronal activity. *J. Neurochem.* 139 (Suppl. 2) 115–125. 10.1111/jnc.13486 26968531 PMC5018236

[B17] Bordone M. P. Salman M. M. Titus H. E. Amini E. Andersen J. V. Chakraborti B. (2019). The energetic brain – a review from students to students. *J. Neurochem.* 151 139–165. 10.1111/jnc.14829 31318452

[B18] Bristow M. S. Simon J. E. Brown R. A. Eliasziw M. Hill M. D. Coutts S. B. (2005). MR perfusion and diffusion in acute ischemic stroke: human gray and white matter have different thresholds for infarction. *J. Cereb. Blood Flow Metab.* 25 1280–1287. 10.1038/sj.jcbfm.9600135 15889043

[B19] Bruick R. K. (2000). Expression of the gene encoding the proapoptotic Nip3 protein is induced by hypoxia. *Proc. Natl. Acad. Sci. U.S.A.* 97 9082–9087. 10.1073/pnas.97.16.9082 10922063 PMC16825

[B20] Bueler H. (2010). Mitochondrial dynamics, cell death and the pathogenesis of Parkinson’s disease. *Apoptosis* 15 1336–1353. 10.1007/s10495-010-0465-0 20131004

[B21] Campbell B. C. V. De Silva D. A. Macleod M. R. Coutts S. B. Schwamm L. H. Davis S. M. (2019). Ischaemic stroke. *Nat. Rev. Dis. Prim.* 5:70. 10.1038/s41572-019-0118-8 31601801

[B22] Cardoso A. R. Queliconi B. B. Kowaltowski A. J. (2010). Mitochondrial ion transport pathways: role in metabolic diseases. *Biochim. Biophys. Acta* 1797 832–838. 10.1016/j.bbabio.2009.12.017 20044972

[B23] Chan D. C. (2020). Mitochondrial dynamics and its involvement in disease. *Annu. Rev. Pathol.* 15 235–259. 10.1146/annurev-pathmechdis-012419-032711 31585519

[B24] Chang C. Y. Liang M. Z. Chen L. (2019). Current progress of mitochondrial transplantation that promotes neuronal regeneration. *Transl. Neurodegener.* 8:17. 10.1186/s40035-019-0158-8 31210929 PMC6567446

[B25] Chen Y. Liu Y. Dorn G. W. II (2011). Mitochondrial fusion is essential for organelle function and cardiac homeostasis. *Circ. Res.* 109 1327–1331. 10.1161/CIRCRESAHA.111.258723 22052916 PMC3237902

[B26] Cheng J. Korte N. Nortley R. Sethi H. Tang Y. Attwell D. (2018). Targeting pericytes for therapeutic approaches to neurological disorders. *Acta Neuropathol.* 136 507–523. 10.1007/s00401-018-1893-0 30097696 PMC6132947

[B27] Chimeh U. Zimmerman M. A. Gilyazova N. Li P. A. (2018). B355252, a novel small molecule, confers neuroprotection against cobalt chloride toxicity in mouse hippocampal cells through altering mitochondrial dynamics and limiting autophagy induction. *Int. J. Med. Sci.* 15 1384–1396. 10.7150/ijms.24702 30275767 PMC6158673

[B28] Chiurazzi M. Di Maro M. Cozzolino M. Colantuoni A. (2020). Mitochondrial dynamics and microglia as new targets in metabolism regulation. *Int. J. Mol. Sci.* 21:3450. 10.3390/ijms21103450 32414136 PMC7279384

[B29] Cho M. H. Kim D. H. Choi J. E. Chang E. J. Seung Y. (2012). Increased phosphorylation of dynamin-related protein 1 and mitochondrial fission in okadaic acid-treated neurons. *Brain Res.* 1454 100–110. 10.1016/j.brainres.2012.03.010 22459049

[B30] Cid-Castro C. Hernandez-Espinosa D. R. Moran J. (2018). ROS as regulators of mitochondrial dynamics in neurons. *Cell. Mol. Neurobiol.* 38 995–1007. 10.1007/s10571-018-0584-7 29687234 PMC11481975

[B31] Cohen M. M. Tareste D. (2018). Recent insights into the structure and function of Mitofusins in mitochondrial fusion. *F1000Research* 7:1983. 10.12688/f1000research.16629.1 30647902 PMC6317495

[B32] Costa V. Giacomello M. Hudec R. Lopreiato R. Ermak G. Lim D. (2010). Mitochondrial fission and cristae disruption increase the response of cell models of Huntington’s disease to apoptotic stimuli. *EMBO Mol. Med.* 2 490–503. 10.1002/emmm.201000102 21069748 PMC3044888

[B33] Crack P. J. Taylor J. M. (2005). Reactive oxygen species and the modulation of stroke. *Free Radic. Biol. Med.* 38 1433–1444. 10.1016/j.freeradbiomed.2005.01.019 15890617

[B34] da Rosa M. S. da Rosa-Junior N. T. Parmeggiani B. Glanzel N. M. de MouraAlvorcem L. Ribeiro R. T. (2020). 3-Hydroxy-3-methylglutaric acid impairs redox and energy homeostasis, mitochondrial dynamics, and endoplasmic reticulum-mitochondria crosstalk in rat brain. *Neurotox. Res.* 37 314–325. 10.1007/s12640-019-00122-x 31721046

[B35] Dorn G. W. II Kitsis R. N. (2015). The mitochondrial dynamism-mitophagy-cell death interactome: multiple roles performed by members of a mitochondrial molecular ensemble. *Circ. Res.* 116 167–182. 10.1161/circresaha.116.303554 25323859 PMC4282600

[B36] Doyle K. P. Simon R. P. Stenzel-Poore M. P. (2008). Mechanisms of ischemic brain damage. *Neuropharmacology* 55 310–318. 10.1016/j.neuropharm.2008.01.005 18308346 PMC2603601

[B37] Droge W. (2002). Free radicals in the physiological control of cell function. *Physiol. Rev.* 82 47–95. 10.1152/physrev.00018.2001 11773609

[B38] Ducommun S. Deak M. Sumpton D. Ford R. J. Galindo A. N. Kussmann M. (2015). Motif affinity and mass spectrometry proteomic approach for the discovery of cellular AMPK targets: identification of mitochondrial fission factor as a new AMPK substrate. *Cell Signal* 27 978–988. 10.1016/j.cellsig.2015.02.008 25683918

[B39] Elmore S. (2007). Apoptosis: a review of programmed cell death. *Toxicol. Pathol.* 35 495–516. 10.1080/01926230701320337 17562483 PMC2117903

[B40] Falkowska A. Gutowska I. Goschorska M. Nowacki P. Chlubek D. Baranowska-Bosiacka I. (2015). Energy metabolism of the brain, including the cooperation between astrocytes and neurons, especially in the context of glycogen metabolism. *Int. J. Mol. Sci.* 16 25959–25981. 10.3390/ijms161125939 26528968 PMC4661798

[B41] Frank S. Gaume B. Bergmann-Leitner E. S. Leitner W. W. Robert E. G. Catez F. (2001). The role of dynamin-related protein 1, a mediator of mitochondrial fission, in apoptosis. *Dev. Cell* 1 515–525. 10.1016/s1534-5807(01)00055-7 11703942

[B42] Fricker M. Tolkovsky A. M. Borutaite V. Coleman M. Brown G. C. (2018). Neuronal cell death. *Physiol. Rev.* 98 813–880. 10.1152/physrev.00011.2017 29488822 PMC5966715

[B43] Friedman J. R. Lackner L. L. West M. DiBenedetto J. R. Nunnari J. Voeltz G. K. (2011). ER tubules mark sites of mitochondrial division. *Science* 334 358–362. 10.1126/science.1207385 21885730 PMC3366560

[B44] Fu M. St-Pierre P. Shankar J. Wang P. T. Joshi B. Nabi I. R. (2013). Regulation of mitophagy by the Gp78 E3 ubiquitin ligase. *Mol. Biol. Cell* 24 1153–1162. 10.1091/mbc.E12-08-0607 23427266 PMC3623636

[B45] GBD 2016 Stroke Collaborators (2019). Global, regional, and national burden of stroke, 1990-2016: a systematic analysis for the Global Burden of Disease Study 2016. *Lancet Neurol.* 18 439–458. 10.1016/S1474-4422(19)30034-1 30871944 PMC6494974

[B46] Gegg M. E. Cooper J. M. Chau K. Y. Rojo M. Schapira A. H. Taanman J. W. (2010). Mitofusin 1 and mitofusin 2 are ubiquitinated in a PINK1/parkin-dependent manner upon induction of mitophagy. *Hum. Mol. Genet.* 19 4861–4870. 10.1093/hmg/ddq419 20871098 PMC3583518

[B47] Gobel J. Engelhardt E. Pelzer P. Sakthivelu V. Jahn H. M. Jevtic M. (2020). Mitochondria-endoplasmic reticulum contacts in reactive astrocytes promote vascular remodeling. *Cell Metab.* 31 791–808.e8. 10.1016/j.cmet.2020.03.005 32220306 PMC7139200

[B48] Gomes L. C. Di Benedetto G. Scorrano L. (2011). During autophagy mitochondria elongate, are spared from degradation and sustain cell viability. *Nat. Cell Biol.* 13 589–598. 10.1038/ncb2220 21478857 PMC3088644

[B49] Green D. R. Reed J. C. (1998). Mitochondria and apoptosis. *Science* 281 1309–1312. 10.1126/science.281.5381.1309 9721092

[B50] Grohm J. Kim S. W. Mamrak U. Tobaben S. Cassidy-Stone A. Nunnari J. (2012). Inhibition of Drp1 provides neuroprotection in vitro and in vivo. *Cell Death Differ.* 19 1446–1458. 10.1038/cdd.2012.18 22388349 PMC3422469

[B51] Guan R. Q. Zou W. Dai X. H. Yu X. P. Liu H. Chen Q. X. (2018). Mitophagy, a potential therapeutic target for stroke. *J. Biomed. Sci.* 25:87. 10.1186/s12929-018-0487-4 30501621 PMC6271612

[B52] Guo Y. Li D. Zhang S. Yang Y. Liu J. J. Wang X. (2018). Visualizing intracellular organelle and cytoskeletal interactions at nanoscale resolution on millisecond timescales. *Cell* 175 1430–1442.e17. 10.1016/j.cell.2018.09.057 30454650

[B53] Gustafsson A. B. Dorn G. W. (2019). Evolving and expanding the roles of mitophagy as a homeostatic and pathogenic process. *Physiol. Rev.* 99 853–892. 10.1152/physrev.00005.2018 30540226 PMC6442924

[B54] Halestrap A. P. Woodfield K. Y. Connern C. P. (1997). Oxidative stress, thiol reagents, and membrane potential modulate the mitochondrial permeability transition by affecting nucleotide binding to the adenine nucleotide translocase. *J. Biol. Chem.* 272 3346–3354. 10.1074/jbc.272.6.3346 9013575

[B55] Harris J. J. Jolivet R. Attwell D. (2012). Synaptic energy use and supply. *Neuron* 75 762–777. 10.1016/j.neuron.2012.08.019 22958818

[B56] Herrero-Mendez A. Almeida A. Fernandez E. Maestre C. Moncada S. Bolanos J. P. (2009). The bioenergetic and antioxidant status of neurons is controlled by continuous degradation of a key glycolytic enzyme by APC/C-Cdh1. *Nat. Cell Biol.* 11 747–752. 10.1038/ncb1881 19448625

[B57] Hofmeijer J. van Putten M. J. (2012). Ischemic cerebral damage: an appraisal of synaptic failure. *Stroke* 43 607–615. 10.1161/STROKEAHA.111.632943 22207505

[B58] Hoppins S. (2014). The regulation of mitochondrial dynamics. *Curr. Opin. Cell Biol.* 29 46–52. 10.1016/j.ceb.2014.03.005 24747170

[B59] Huang P. J. Kuo C. C. Lee H. C. Shen C. I. Cheng F. C. Wu S. F. (2016). Transferring xenogenic mitochondria provides neural protection against ischemic stress in ischemic rat brains. *Cell Transplant.* 25 913–927. 10.3727/096368915X689785 26555763

[B60] Hurst S. Hoek J. Sheu S. S. (2017). Mitochondrial Ca(2+) and regulation of the permeability transition pore. *J. Bioenerget. Biomembr.* 49 27–47. 10.1007/s10863-016-9672-x 27497945 PMC5393273

[B61] Hwang J. A. Shin N. Shin H. J. Yin Y. Kwon H. H. Park H. (2020). Protective effects of ShcA protein silencing for photothrombotic cerebral infarction. *Transl. Stroke Res.* 10.1007/s12975-020-00874-1 33242144

[B62] Iadecola C. (2017). The neurovascular unit coming of age: a journey through neurovascular coupling in health and disease. *Neuron* 96 17–42. 10.1016/j.neuron.2017.07.030 28957666 PMC5657612

[B63] Jeong S. Y. Seol D. W. (2008). The role of mitochondria in apoptosis. *BMB Rep.* 41 11–22. 10.5483/bmbrep.2008.41.1.011 18304445

[B64] Jurgensmeier J. M. Xie Z. Deveraux Q. Ellerby L. Bredesen D. Reed J. C. (1998). Bax directly induces release of cytochrome c from isolated mitochondria. *Proc. Natl. Acad. Sci. U.S.A.* 95 4997–5002. 10.1073/pnas.95.9.4997 9560217 PMC20202

[B65] Kim T. Mehta S. L. Morris-Blanco K. C. Chokkalla A. K. Chelluboina B. Lopez M. (2018). The microRNA miR-7a-5p ameliorates ischemic brain damage by repressing alpha-synuclein. *Sci. Signal.* 11:eaat4285. 10.1126/scisignal.aat4285 30538177 PMC7005928

[B66] Kinnally K. W. Peixoto P. M. Ryu S. Y. Dejean L. M. (2011). Is mPTP the gatekeeper for necrosis, apoptosis, or both? *BBA Mol. Cell Res.* 1813 616–622. 10.1016/j.bbamcr.2010.09.013 20888866 PMC3050112

[B67] Kornfeld O. S. Qvit N. Haileselassie B. Shamloo M. Bernardi P. Mochly-Rosen D. (2018). Interaction of mitochondrial fission factor with dynamin related protein 1 governs physiological mitochondrial function in vivo. *Sci. Rep.* 8:14034. 10.1038/s41598-018-32228-1 30232469 PMC6145916

[B68] Koshiba T. Detmer S. A. Kaiser J. T. Chen H. McCaffery J. M. Chan D. C. (2004). Structural basis of mitochondrial tethering by mitofusin complexes. *Science* 305 858–862. 10.1126/science.1099793 15297672

[B69] Kwong J. Q. Lu X. Correll R. N. Schwanekamp J. A. Vagnozzi R. J. Sargent M. A. (2015). The mitochondrial calcium uniporter selectively matches metabolic output to acute contractile stress in the heart. *Cell Rep.* 12 15–22. 10.1016/j.celrep.2015.06.002 26119742 PMC4497842

[B70] Lampert M. A. Orogo A. M. Najor R. H. Hammerling B. C. Leon L. J. Wang B. J. (2019). BNIP3L/NIX and FUNDC1-mediated mitophagy is required for mitochondrial network remodeling during cardiac progenitor cell differentiation. *Autophagy* 15 1182–1198. 10.1080/15548627.2019.1580095 30741592 PMC6613840

[B71] Leigh R. Knutsson L. Zhou J. van Zijl P. C. (2018). Imaging the physiological evolution of the ischemic penumbra in acute ischemic stroke. *J. Cereb. Blood Flow Metab.* 38 1500–1516. 10.1177/0271678X17700913 28345479 PMC6125975

[B72] Lewis S. C. Uchiyama L. F. Nunnari J. (2016). ER-mitochondria contacts couple mtDNA synthesis with mitochondrial division in human cells. *Science* 353:aaf5549. 10.1126/science.aaf5549 27418514 PMC5554545

[B73] Lindsay M. P. Norrving B. Sacco R. L. Brainin M. Hacke W. Martins S. (2019). World stroke organization (WSO): global stroke fact sheet 2019. *Int. J. Stroke* 14 806–817. 10.1177/1747493019881353 31658892

[B74] Lunt S. Y. Vander Heiden M. G. (2011). Aerobic glycolysis: meeting the metabolic requirements of cell proliferation. *Annu. Rev. Cell Dev. Biol.* 27 441–464. 10.1146/annurev-cellbio-092910-154237 21985671

[B75] Magistretti P. J. Allaman I. (2018). Lactate in the brain: from metabolic end-product to signalling molecule. *Nat. Rev. Neurosci.* 19 235–249. 10.1038/nrn.2018.19 29515192

[B76] Mandal A. Drerup C. M. (2019). Axonal transport and mitochondrial function in neurons. *Front. Cell. Neurosci.* 13:373. 10.3389/fncel.2019.00373 31447650 PMC6696875

[B77] Matsumoto S. Friberg H. Ferrand-Drake M. Wieloch T. (1999). Blockade of the mitochondrial permeability transition pore diminishes infarct size in the rat after transient middle cerebral artery occlusion. *J. Cereb. Blood Flow Metab.* 19 736–741. 10.1097/00004647-199907000-00002 10413027

[B78] Mergenthaler P. Lindauer U. Dienel G. A. Meisel A. (2013). Sugar for the brain: the role of glucose in physiological and pathological brain function. *Trends Neurosci.* 36 587–597. 10.1016/j.tins.2013.07.001 23968694 PMC3900881

[B79] Modi J. Menzie-Suderam J. Xu H. Trujillo P. Medley K. Marshall M. L. (2020). Mode of action of granulocyte-colony stimulating factor (G-CSF) as a novel therapy for stroke in a mouse model. *J. Biomed. Sci.* 27:19. 10.1186/s12929-019-0597-7 31907023 PMC6943893

[B80] Montessuit S. Somasekharan S. P. Terrones O. Lucken-Ardjomande S. Herzig S. Schwarzenbacher R. (2010). Membrane remodeling induced by the dynamin-related protein Drp1 stimulates Bax oligomerization. *Cell* 142 889–901. 10.1016/j.cell.2010.08.017 20850011 PMC4115189

[B81] Moskowitz M. A. Lo E. H. Iadecola C. (2010). The science of stroke: mechanisms in search of treatments. *Neuron* 67 181–198. 10.1016/j.neuron.2010.07.002 20670828 PMC2957363

[B82] Neuspiel M. Zunino R. Gangaraju S. Rippstein P. McBride H. (2005). Activated mitofusin 2 signals mitochondrial fusion, interferes with Bax activation, and reduces susceptibility to radical induced depolarization. *J. Biol. Chem.* 280 25060–25070. 10.1074/jbc.M501599200 15878861

[B83] Nguyen D. Alavi M. V. Kim K. Y. Kang T. Scott R. T. Noh Y. H. (2011). A new vicious cycle involving glutamate excitotoxicity, oxidative stress and mitochondrial dynamics. *Cell Death Dis.* 2:e240. 10.1038/cddis.2011.117 22158479 PMC3252734

[B84] Ni X. X. Nie J. Xie Q. Y. Yu R. H. Su L. Liu Z. F. (2020). Protective effects of hyperbaric oxygen therapy on brain injury by regulating the phosphorylation of Drp1 through ROS/PKC pathway in heatstroke rats. *Cell. Mol. Neurobiol.* 40 1253–1269. 10.1007/s10571-020-00811-8 32043174 PMC11448848

[B85] Norbury C. J. Hickson I. D. (2001). Cellular responses to DNA damage. *Annu. Rev. Pharmacol. Toxicol.* 41 367–401. 10.1146/annurev.pharmtox.41.1.367 11264462

[B86] Nortley R. Attwell D. (2017). Control of brain energy supply by astrocytes. *Curr. Opin. Neurobiol.* 47 80–85. 10.1016/j.conb.2017.09.012 29054039

[B87] Nunnari J. (2007). The machines that divide and fuse mitochondria. *FASEB J.* 21:A96. 10.1146/annurev.biochem.76.071905.090048 17362197

[B88] O’Donnell J. C. Jackson J. G. Robinson M. B. (2016). Transient oxygen/glucose deprivation causes a delayed loss of mitochondria and increases spontaneous calcium signaling in astrocytic processes. *J. Neurosci.* 36 7109–7127. 10.1523/Jneurosci.4518-15.2016 27383588 PMC4938859

[B89] Ong S. B. Subrayan S. Lim S. Y. Yellon D. M. Davidson S. M. Hausenloy D. J. (2010). Inhibiting mitochondrial fission protects the heart against ischemia/reperfusion injury. *Circulation* 121 2012–2022. 10.1161/CIRCULATIONAHA.109.906610 20421521

[B90] Pagliuso A. Cossart P. Stavru F. (2018). The ever-growing complexity of the mitochondrial fission machinery. *Cell. Mol. Life Sci.* 75 355–374. 10.1007/s00018-017-2603-0 28779209 PMC5765209

[B91] Papanicolaou K. N. Khairallah R. J. Ngoh G. A. Chikando A. Luptak I. O’Shea K. M. (2011). Mitofusin-2 maintains mitochondrial structure and contributes to stress-induced permeability transition in cardiac myocytes. *Mol. Cell. Biol.* 31 1309–1328. 10.1128/MCB.00911-10 21245373 PMC3067905

[B92] Park J. Choi H. Min J. S. Park S. J. Kim J. H. Park H. J. (2013). Mitochondrial dynamics modulate the expression of pro-inflammatory mediators in microglial cells. *J. Neurochem.* 127 221–232. 10.1111/jnc.12361 23815397

[B93] Pivovarova N. B. Nguyen H. V. Winters C. A. Brantner C. A. Smith C. L. Andrews S. B. (2004). Excitotoxic calcium overload in a subpopulation of mitochondria triggers delayed death in hippocampal neurons. *J. Neurosci.* 24 5611–5622. 10.1523/JNEUROSCI.0531-04.2004 15201334 PMC6729327

[B94] Powers W. J. Rabinstein A. A. Ackerson T. Adeoye O. M. Bambakidis N. C. Becker K. (2018). 2018 Guidelines for the early management of patients with acute ischemic stroke: a guideline for healthcare professionals from the American heart association/American stroke association. *Stroke* 49 e46–e110. 10.1161/STR.0000000000000158 29367334

[B95] Prabhakaran S. Ruff I. Bernstein R. A. (2015). Acute stroke intervention: a systematic review. *JAMA* 313 1451–1462. 10.1001/jama.2015.3058 25871671

[B96] Pushie M. J. Crawford A. M. Sylvain N. J. Hou H. Hackett M. J. George G. N. (2018). Revealing the penumbra through imaging elemental markers of cellular metabolism in an ischemic stroke model. *ACS Chem. Neurosci.* 9 886–893. 10.1021/acschemneuro.7b00382 29370523

[B97] Qi X. Qvit N. Su Y. C. Mochly-Rosen D. (2013). A novel Drp1 inhibitor diminishes aberrant mitochondrial fission and neurotoxicity. *J. Cell Sci.* 126 789–802. 10.1242/jcs.114439 23239023 PMC3619809

[B98] Qin Y. Li A. Liu B. Jiang W. Gao M. Tian X. (2020). Mitochondrial fusion mediated by fusion promotion and fission inhibition directs adult mouse heart function toward a different direction. *FASEB J.* 34 663–675. 10.1096/fj.201901671R 31914595

[B99] Quintana D. D. Garcia J. A. Sarkar S. N. Jun S. J. Engler-Chiurazzi E. B. Russell A. E. (2019). Hypoxia-reoxygenation of primary astrocytes results in a redistribution of mitochondrial size and mitophagy. *Mitochondrion* 47 244–255. 10.1016/j.mito.2018.12.004 30594729 PMC6980114

[B100] Radak D. Katsiki N. Resanovic I. Jovanovic A. Sudar-Milovanovic E. Zafirovic S. (2017). Apoptosis and acute brain ischemia in ischemic stroke. *Curr. Vasc. Pharmacol.* 15 115–122. 10.2174/1570161115666161104095522 27823556

[B101] Rao G. Murphy B. Dey A. Dwivedi S. K. D. Zhang Y. Roy R. V. (2020). Cystathionine beta synthase regulates mitochondrial dynamics and function in endothelial cells. *FASEB J.* 34 9372–9392. 10.1096/fj.202000173R 32463541 PMC7675787

[B102] Rappold P. M. Cui M. Grima J. C. Fan R. Z. de Mesy-Bentley K. L. Chen L. (2014). Drp1 inhibition attenuates neurotoxicity and dopamine release deficits in vivo. *Nat. Commun.* 5:5244. 10.1038/ncomms6244 25370169 PMC4223875

[B103] Rigoulet M. Bouchez C. L. Paumard P. Ransac S. Cuvellier S. Duvezin-Caubet S. (2020). Cell energy metabolism: an update. *Biochim. Biophys. Acta Bioenerget.* 1861:148276. 10.1016/j.bbabio.2020.148276 32717222

[B104] Rinholm J. E. Vervaeke K. Tadross M. R. Tkachuk A. N. Kopek B. G. Brown T. A. (2016). Movement and structure of mitochondria in oligodendrocytes and their myelin sheaths. *Glia* 64 810–825. 10.1002/glia.22965 26775288

[B105] Rodger C. E. McWilliams T. G. Ganley I. G. (2018). Mammalian mitophagy – from in vitro molecules to in vivo models. *FEBS J.* 285 1185–1202. 10.1111/febs.14336 29151277 PMC5947125

[B106] Roy M. Reddy P. H. Iijima M. Sesaki H. (2015). Mitochondrial division and fusion in metabolism. *Curr. Opin. Cell Biol.* 33 111–118. 10.1016/j.ceb.2015.02.001 25703628 PMC4380865

[B107] Ruiz A. Quintela-López T. Sánchez-Gómez M. V. Gaminde-Blasco A. Alberdi E. Matute C. (2020). Mitochondrial division inhibitor 1 disrupts oligodendrocyte Ca(2+)homeostasis and mitochondrial function. *Glia* 68 1743–1756. 10.1002/glia.23802 32060978

[B108] Sarrafzadeh A. S. Nagel A. Czabanka M. Denecke T. Vajkoczy P. Plotkin M. (2010). Imaging of hypoxic-ischemic penumbra with F-18-fluoromisonidazole PET/CT and measurement of related cerebral metabolism in aneurysmal subarachnoid hemorrhage. *J. Cereb. Blood Flow Metab.* 30 36–45. 10.1038/jcbfm.2009.199 19773799 PMC2949093

[B109] Scheffler I. E. (2001). Mitochondria make a come back. *Adv. Drug Deliv. Rev.* 49 3–26. 10.1016/S0169-409x(01)00123-5 11377800

[B110] Sekerdag E. Solaroglu I. Gursoy-Ozdemir Y. (2018). Cell death mechanisms in stroke and novel molecular and cellular treatment options. *Curr. Neuropharmacol.* 16 1396–1415. 10.2174/1570159X16666180302115544 29512465 PMC6251049

[B111] Sharp W. W. Fang Y. H. Han M. Zhang H. J. Hong Z. Banathy A. (2014). Dynamin-related protein 1 (Drp1)-mediated diastolic dysfunction in myocardial ischemia-reperfusion injury: therapeutic benefits of Drp1 inhibition to reduce mitochondrial fission. *FASEB J.* 28 316–326. 10.1096/fj.12-226225 24076965 PMC3868827

[B112] Sheng Z. H. (2014). Mitochondrial trafficking and anchoring in neurons: new insight and implications. *J. Cell Biol.* 204 1087–1098. 10.1083/jcb.201312123 24687278 PMC3971748

[B113] Sokoloff L. (1981). Localization of functional activity in the central nervous system by measurement of glucose utilization with radioactive deoxyglucose. *J. Cereb. Blood Flow Metab.* 1 7–36. 10.1038/jcbfm.1981.4 7035471

[B114] Sokoloff L. Reivich M. Kennedy C. Des Rosiers M. H. Patlak C. S. Pettigrew K. D. (1977). The [14C]deoxyglucose method for the measurement of local cerebral glucose utilization: theory, procedure, and normal values in the conscious and anesthetized albino rat. *J. Neurochem.* 28 897–916. 10.1111/j.1471-4159.1977.tb10649.x 864466

[B115] Song T. Liu J. Tao X. Deng J. G. (2014). Protection effect of atorvastatin in cerebral ischemia-reperfusion injury rats by blocking the mitochondrial permeability transition pore. *Genet. Mol. Res.* 13 10632–10642. 10.4238/2014.December.18.5 25526184

[B116] Song Z. Chen H. Fiket M. Alexander C. Chan D. C. (2007). OPA1 processing controls mitochondrial fusion and is regulated by mRNA splicing, membrane potential, and Yme1L. *J. Cell Biol.* 178 749–755. 10.1083/jcb.200704110 17709429 PMC2064540

[B117] Sowter H. M. Ratcliffe P. J. Watson P. Greenberg A. H. Harris A. L. (2001). HIF-1-dependent regulation of hypoxic induction of the cell death factors BNIP3 and NIX in human tumors. *Cancer Res.* 61 6669–6673. 11559532

[B118] Sprenger H. G. Langer T. (2019). The good and the bad of mitochondrial breakups. *Trends Cell Biol.* 29 888–900. 10.1016/j.tcb.2019.08.003 31495461

[B119] Susin S. A. Lorenzo H. K. Zamzami N. Marzo I. Snow B. E. Brothers G. M. (1999). Molecular characterization of mitochondrial apoptosis-inducing factor. *Nature* 397 441–446. 10.1038/17135 9989411

[B120] Tilokani L. Nagashima S. Paupe V. Prudent J. (2018). Mitochondrial dynamics: overview of molecular mechanisms. *Essays Biochem.* 62 341–360. 10.1042/EBC20170104 30030364 PMC6056715

[B121] Toyama E. Q. Herzig S. Courchet J. Lewis T. L. Loson O. C. Hellberg K. (2016). AMP-activated protein kinase mediates mitochondrial fission in response to energy stress. *Science* 351 275–281. 10.1126/science.aab4138 26816379 PMC4852862

[B122] Tucker L. D. Lu Y. Dong Y. Yang L. Li Y. Zhao N. (2018). Photobiomodulation therapy attenuates hypoxic-ischemic injury in a neonatal rat model. *J. Mol. Neurosci.* 65 514–526. 10.1007/s12031-018-1121-3 30032397 PMC6109412

[B123] Um J. H. Yun J. (2017). Emerging role of mitophagy in human diseases and physiology. *BMB Rep.* 50 299–307. 10.5483/BMBRep.2017.50.6.056 28366191 PMC5498140

[B124] Varanita T. Soriano M. E. Romanello V. Zaglia T. Quintana-Cabrera R. Semenzato M. (2015). The Opa1-dependent mitochondrial cristae remodeling pathway controls atrophic, apoptotic, and ischemic tissue damage. *Cell Metab.* 21 834–844. 10.1016/j.cmet.2015.05.007 26039448 PMC4457892

[B125] Wai T. Langer T. (2016). Mitochondrial dynamics and metabolic regulation. *Trends Endocrinol. Metab.* 27 105–117. 10.1016/j.tem.2015.12.001 26754340

[B126] Wang J. Zhu P. Li R. Ren J. Zhou H. (2020). Fundc1-dependent mitophagy is obligatory to ischemic preconditioning-conferred renoprotection in ischemic AKI via suppression of Drp1-mediated mitochondrial fission. *Redox Biol.* 30:101415. 10.1016/j.redox.2019.101415 31901590 PMC6940662

[B127] Wang W. Z. Zhang F. Li L. Tang F. Q. Siedlak S. L. Fujioka H. (2015). MFN2 couples glutamate excitotoxicity and mitochondrial dysfunction in motor neurons. *J. Biol. Chem.* 290 168–182. 10.1074/jbc.M114.617167 25416777 PMC4281719

[B128] Wang Y. Liu G. Hong D. D. Chen F. H. Ji X. M. Cao G. D. (2016). White matter injury in ischemic stroke. *Prog. Neurobiol.* 141 45–60. 10.1016/j.pneurobio.2016.04.00 27090751 PMC5677601

[B129] Wang Y. Zhou L. Guo J. Wang Y. Yang Y. Peng Q. (2020). Secular trends of stroke incidence and mortality in China, 1990 to 2016: the global burden of disease study 2016. *J. Stroke Cerebrovasc. Dis.* 29:104959. 10.1016/j.jstrokecerebrovasdis.2020.104959 32689583

[B130] Wang Z. G. Jiang H. Chen S. Du F. H. Wang X. D. (2012). The mitochondrial phosphatase PGAM5 functions at the convergence point of multiple necrotic death pathways. *Cell* 148 228–243. 10.1016/j.cell.2011.11.030 22265414

[B131] Wei N. Pu Y. Yang Z. Pan Y. Liu L. (2019). Therapeutic effects of melatonin on cerebral ischemia reperfusion injury: role of Yap-OPA1 signaling pathway and mitochondrial fusion. *Biomed. Pharmacother.* 110 203–212. 10.1016/j.biopha.2018.11.060 30476721

[B132] Westermann B. (2010). Mitochondrial fusion and fission in cell life and death. *Nat. Rev. Mol. Cell Biol.* 11 872–884. 10.1038/nrm3013 21102612

[B133] White K. E. Davies V. J. Hogan V. E. Piechota M. J. Nichols P. P. Turnbull D. M. (2009). OPA1 deficiency associated with increased autophagy in retinal ganglion cells in a murine model of dominant optic atrophy. *Invest. Ophth. Vis. Sci.* 50 2567–2571. 10.1167/iovs.08-2913 19234344

[B134] Williams J. A. Ding W. X. (2018). Mechanisms, pathophysiological roles and methods for analyzing mitophagy – recent insights. *Biol. Chem.* 399 147–178. 10.1515/hsz-2017-0228 28976892 PMC5835338

[B135] Wu B. Luo H. Zhou X. Cheng C. Y. Lin L. Liu B. L. (2017). Succinate-induced neuronal mitochondrial fission and hexokinase II malfunction in ischemic stroke: therapeutical effects of kaempferol. *BBA Mol. Basis Dis.* 1863 2307–2318. 10.1016/j.bbadis.2017.06.011 28634116

[B136] Wu Q. Gao C. Wang H. Zhang X. Li Q. Gu Z. (2018). Mdivi-1 alleviates blood-brain barrier disruption and cell death in experimental traumatic brain injury by mitigating autophagy dysfunction and mitophagy activation. *Int. J. Biochem. Cell Biol.* 94 44–55. 10.1016/j.biocel.2017.11.007 29174311

[B137] Xie L. L. Shi F. Tan Z. Li Y. Bode A. M. Cao Y. (2018). Mitochondrial network structure homeostasis and cell death. *Cancer Sci.* 109 3686–3694. 10.1111/cas.13830 30312515 PMC6272111

[B138] Yaghi S. Willey J. Z. Cucchiara B. Goldstein J. N. Gonzales N. R. Khatri P. (2017). Treatment and outcome of hemorrhagic transformation after intravenous alteplase in acute ischemic stroke: a scientific statement for healthcare professionals from the American heart association/American stroke association. *Stroke* 48 e343–e361. 10.1161/STR.0000000000000152 29097489

[B139] Yoo S. M. Jung Y. K. (2018). A molecular approach to mitophagy and mitochondrial dynamics. *Mol. Cells* 41 18–26. 10.14348/molcells.2018.2277 29370689 PMC5792708

[B140] Youle R. J. van der Bliek A. M. (2012). Mitochondrial fission, fusion, and stress. *Science* 337 1062–1065. 10.1126/science.1219855 22936770 PMC4762028

[B141] Yu B. Ma J. Li J. Wang D. Z. Wang Z. G. Wang S. S. (2020). Mitochondrial phosphatase PGAM5 modulates cellular senescence by regulating mitochondrial dynamics. *Nat. Commun.* 11:2549. 10.1038/s41467-020-16312-7 32439975 PMC7242393

[B142] Zenonos G. Kim J. E. (2010). 18F-fluoromisonidazole PET/CT and related gauges of cerebral metabolism identify salvageable tissue in ischemic penumbra following aneurysmal subarachnoid hemorrhage. *Neurosurgery* 66 N13–N15. 10.1227/01.neu.0000367839.75141.e6 20087119

[B143] Zhang C. Wang J. Zhu J. Chen Y. Han X. (2020). Microcystin-leucine-arginine induced neurotoxicity by initiating mitochondrial fission in hippocampal neurons. *Sci. Total Environ.* 703:134702. 10.1016/j.scitotenv.2019.134702 31753492

[B144] Zhang H. S. Liu C. D. Zheng M. C. Zhao H. T. Liu X. J. (2020). Propofol alleviates hypoxic neuronal injury by inhibiting high levels of mitochondrial fusion and fission. *Eur. Rev. Med. Pharmacol. Sci.* 24 9650–9657. 10.26355/eurrev_202009_23054 33015809

[B145] Zhang K. Li H. Song Z. (2014). Membrane depolarization activates the mitochondrial protease OMA1 by stimulating self-cleavage. *EMBO Rep.* 15 576–585. 10.1002/embr.201338240 24719224 PMC4210089

[B146] Zhang L. He Z. Zhang Q. Wu Y. Yang X. Niu W. (2014). Exercise pretreatment promotes mitochondrial dynamic protein OPA1 expression after cerebral ischemia in rats. *Int. J. Mol. Sci.* 15 4453–4463. 10.3390/ijms15034453 24633199 PMC3975407

[B147] Zhang Y. K. Sun R. J. Li X. L. Fang W. H. (2020). Porcine circovirus 2 induction of ROS is responsible for mitophagy in PK-15 cells via activation of Drp1 phosphorylation. *Viruses Basel* 12:289. 10.3390/v12030289 32155766 PMC7150875

[B148] Zhang Z. Yu J. (2018). Nurr1 exacerbates cerebral ischemia-reperfusion injury via modulating YAP-INF2-mitochondrial fission pathways. *Int. J. Biochem. Cell Biol.* 104 149–160. 10.1016/j.biocel.2018.09.014 30267803

[B149] Zhao Q. Wang S. Li Y. Wang P. Li S. Guo Y. (2013). The role of the mitochondrial calcium uniporter in cerebral ischemia/reperfusion injury in rats involves regulation of mitochondrial energy metabolism. *Mol. Med. Rep.* 7 1073–1080. 10.3892/mmr.2013.1321 23426506

[B150] Zhao Y. X. Cui M. Chen S. F. Dong Q. Liu X. Y. (2014). Amelioration of ischemic mitochondrial injury and Bax-dependent outer membrane permeabilization by Mdivi-1. *CNS Neurosci. Ther.* 20 528–538. 10.1111/cns.12266 24712408 PMC6493009

[B151] Zhou K. Chen J. Wu J. Wu Q. Jia C. Xu Y. X. Z. (2019a). Atractylenolide III ameliorates cerebral ischemic injury and neuroinflammation associated with inhibiting JAK2/STAT3/Drp1-dependent mitochondrial fission in microglia. *Phytomedicine* 59:152922. 10.1016/j.phymed.2019.152922 30981186

[B152] Zhou K. Wu J. Y. Chen J. Zhou Y. Chen X. L. Wu Q. Y. (2019b). Schaftoside ameliorates oxygen glucose deprivation-induced inflammation associated with the TLR4/Myd88/Drp1-related mitochondrial fission in BV2 microglia cells. *J. Pharmacol. Sci.* 139 15–22. 10.1016/j.jphs.2018.10.012 30528467

[B153] Zhou L. Zhang Q. Zhang P. Sun L. Peng C. Yuan Z. (2017). c-Abl-mediated Drp1 phosphorylation promotes oxidative stress-induced mitochondrial fragmentation and neuronal cell death. *Cell Death Dis.* 8:e3117. 10.1038/cddis.2017.524 29022905 PMC5682686

[B154] Zhou L. Y. Yao M. Tian Z. R. Liu S. F. Song Y. J. Ye J. (2020). Muscone suppresses inflammatory responses and neuronal damage in a rat model of cervical spondylotic myelopathy by regulating Drp1-dependent mitochondrial fission. *J. Neurochem.* 155 154–176. 10.1111/jnc.15011 32215908

